# Teaching Ultrasound-Guided Venous Cannulation to Newly Qualified Doctors

**DOI:** 10.7759/cureus.58295

**Published:** 2024-04-15

**Authors:** Joseph J Gleeson, Hayley Boal, Thomas Sharp, Rebecca Morris

**Affiliations:** 1 Medical Education and Simulation, The Mid Yorkshire Teaching NHS Trust, Wakefield, GBR

**Keywords:** intravenous cannulation, nhs england, post grad medical education, practical skills, ultrasound-guided, near peer teaching, foundation year 1

## Abstract

Background

Venous cannulation is an essential task that allows the intravenous administration of fluids and medications. In the United Kingdom, this task is often performed by newly qualified Foundation Year 1 (FY1) doctors; however, difficulties are commonly encountered. The usage of ultrasound increases the chance of successful cannulation, provided the operator has been trained. Some medical schools now include ultrasound in their undergraduate curricula, though this is far from universal.

Methods

Forty-eight FY1s received a one-hour teaching session on ultrasound-guided venous cannulation, delivered by near-peer Education Fellows. FY1s completed questionnaires immediately after the teaching session, and a follow-up questionnaire three months later.

Findings

44.44% of FY1s felt “fairly” or “very” confident in ultrasound-guided venous cannulation at follow-up, compared to 6.66% before the session. Sixty-three attempts were made in the month before the follow-up survey, compared to six in the month prior to the teaching session. The success rate at follow-up was 60% (38/63), up from 50% (3/6) prior to the session. One third fewer cannulas were escalated to senior doctors (72 vs 48), although there was little change in escalations to anesthetists, from 15 vs 18. FY1s identified the lack of ultrasound machines on the wards as a barrier to using ultrasound-guided venous cannulation more often.

Conclusion

A short, near-peer teaching session can improve FY1s’ confidence, usage, and success rates in ultrasound-guided venous cannulation.

## Introduction

Within the NHS, gaining intravenous access in patients is a fundamental task for newly qualified Foundation Year 1 (FY1) doctors. While FY1s often feel reasonably well-prepared to perform this skill [1], the success rate can be highly variable, even among experienced nurses experienced in venous cannulation [2]. Factors that make cannulation more difficult include previous overuse of veins, obesity, non-visible veins, a history of intravenous drug use, and peripheral vasoconstriction [3,4] - all factors commonly encountered in hospital inpatients. Where the FY1 is unable to gain IV access, they must then seek help from a senior doctor in their team, or the on-call anesthetist. This delays the administration of medications for patients and impacts services elsewhere by taking senior doctors away from other, more complex tasks.

Large studies have concluded that the usage of ultrasound can significantly increase the chance of successful cannulation, especially in patients with otherwise difficult vascular access [5-7]. Training in the skill is required [8], but this training is increasingly being provided, including within undergraduate medical curricula [9-11].

Training staff in ultrasound-guided venous cannulation has been shown to have a positive impact on service provision - for example, one department trained nurses in the skill and saw a significant decrease in the need for PICC and midline placement [12]; another trained foundation doctors and found they regularly used their unit’s machine and felt confident in doing so [13]; and one NHS Trust trained all FY1s in ultrasound and found the FY1s were still confident in, and regularly using the skill several months later [14].

It has been suggested that foundation trainees may be an ideal audience for teaching USS-guided venous cannulation to, given their frequent need to gain access in patients with poor vascular access [15].

STR1DE and ultrasound-guided venous cannulation

Our NHS Trust introduced a new FY1 teaching program - Simulation, Teaching, and Reflection for FY1 Development & Education (STR1DE) - in 2021/22. STR1DE involves FY1s attending six full-day sessions throughout the year and aims to provide practical, useful, and enjoyable teaching to the FY1s. STR1DE is taught by the Trust’s Clinical Fellows in Education & Simulation, who are post-foundation “FY3” and “FY4” junior doctors. As part of the first STR1DE session, FY1s received an hour-long teaching session on ultrasound-guided venous cannulation.

Research questions

A number of research questions were identified: (1) Did the teaching session lead to an increase in confidence following the session, and if so, would this increase in confidence be sustained three months later?; (2) Would the teaching session prompt FY1s to use ultrasound-guided venous cannulation more often on the wards?; (3) If so, would this be correlated with a reduction in calls to specialty seniors and anesthetists for assistance with cannulation?; (4) Would the teaching session lead to an increase in successful attempts at ultrasound-guided venous cannulation?

This article was previously presented as a short communication session on August 29, 2022 at the AMEE 2022 Conference in Lyon.

## Materials and methods

Teaching session design

The session was facilitated by FY3 and FY4 Clinical Teaching Fellows and was therefore near-peer led. Near-peer teaching is recognized to have benefits such as creating a pleasant learning environment and allowing role-modeling of desirable behaviors and skills [16]. This approach has successfully been used in teaching practical skills [17]. We believed this to be a useful approach, as many of the Fellows regularly use ultrasound-guided venous cannulation in their practice, and could therefore role model the usage of the skill to the FY1s in an understandable and realistic way.

The session itself took a multi-step approach. Firstly, there was a small group didactic session briefly covering the indications for use, how to set up the machines, the pros and cons of using in and out-of-plane approaches, and the principles of identifying veins. Next, FY1s used ultrasound machines to locate anatomy on one another.

Once the facilitators were content with FY1s’ skills in identifying veins, they provided the FY1s with Blue Phantom tissue models and cannulas. FY1s then had approximately 40 minutes of independent practice, receiving feedback from facilitators and peers throughout.

Questionnaire design

The teaching session took place when the FY1s had been in post for approximately one month. Immediately following the session, the FY1s were asked to complete an optional feedback questionnaire. Informed consent was given, and all FY1s were free to choose not to respond if they did not want to. The questionnaire asked them to rate their confidence in ultrasound-guided venous cannulation before and after the teaching session; their number of attempts using ultrasound-guided venous cannulation in the month prior to the teaching session and how many of these were successful; and the number of times they had escalated a cannula to a senior doctor in their team, or an anesthetist in the month prior to the teaching session.

The second session of STR1DE took place around three months after the first session. Prior to this session, FY1s were asked to complete a further optional feedback questionnaire, which included the same questions as above.

Data modification

Before the teaching session, there was an overall completion rate of 40/48 (83%), and at follow-up, there was a completion rate of 41/48 (85%). However, for the purposes of comparison and analyzing changes, not all of this data could be used for the following reasons: (1) some FY1s did not complete both surveys; (2) some FY1s did not enter a correct participant ID, meaning their responses could not be compared across the surveys; (3) some FY1s only partially completed the survey. In total, 33/48 (68.75%) of FY1s completed both surveys.

It was also found that a small number of FY1s answered some questions by giving a range (e.g., five-six escalations to seniors), or by writing something such as “many”. For the purpose of consistency, the decision was taken to take the higher of two numbers in a range and to replace the small number of “many” responses with seven (given that six was otherwise the highest number of attempts/escalations, and it was felt that FY1s could reasonably be expected to recall an exact number if it were lower than seven).

Finally, it was decided to exclude FY1s working in certain specialties from the analysis of some of the research questions, namely those FY1s in specialties where US-guided venous cannulation was never used (psychiatry and pediatrics), and those FY1s working on anesthetics/ICU, where the expectations of FY1s, the availability of seniors, and access to ultrasound machine is so different from other FY1s that including their data would be unhelpful.

## Results

Level of confidence

**Figure 1:**
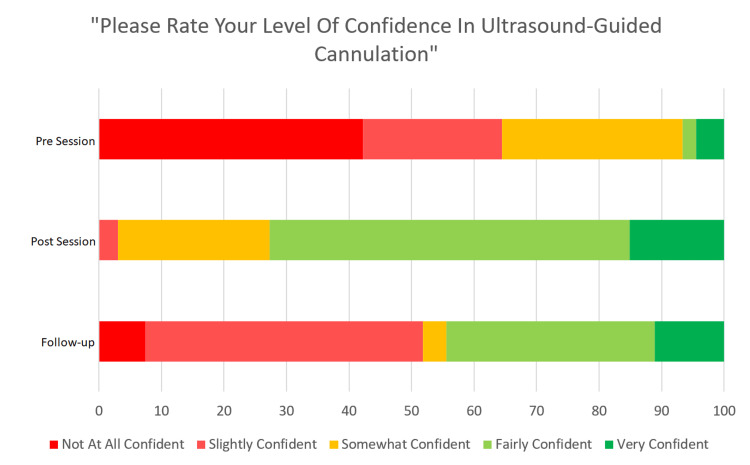
FY1s’ confidence in ultrasound-guided venous cannulation before and immediately after the teaching session, and at follow-up three months later.

FY1s reported much greater confidence in US-guided venous cannulation immediately following the session, with 72% “fairly” or “very” confident after the session compared to 6.66% before the session (Figure 1).

As expected, there was a decrease in confidence from immediately post-session to December (44.44% “fairly” or “very” confident) - nonetheless a large improvement from before the teaching session was sustained.

Usage of ultrasound-guided venous cannulation

**Figure 2:**
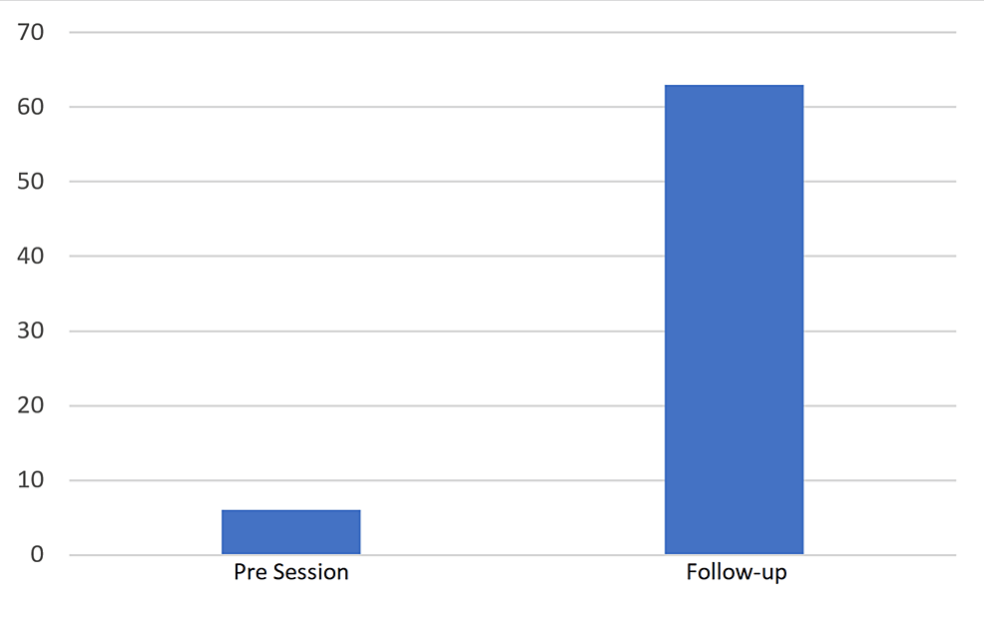
Bar chart comparing the total number of attempts made at ultrasound-guided cannulation in the month before the teaching session and the month prior to follow-up.

FY1s made vastly more attempts at ultrasound-guided venous cannulation three months after the teaching session compared to the month before the teaching session - there were just six attempts (average 0.21 per FY1) made in the month before the teaching session, compared to 63 attempts (average 2.25 per FY1) in the month prior to follow-up (Figure 2).

**Figure 3:**
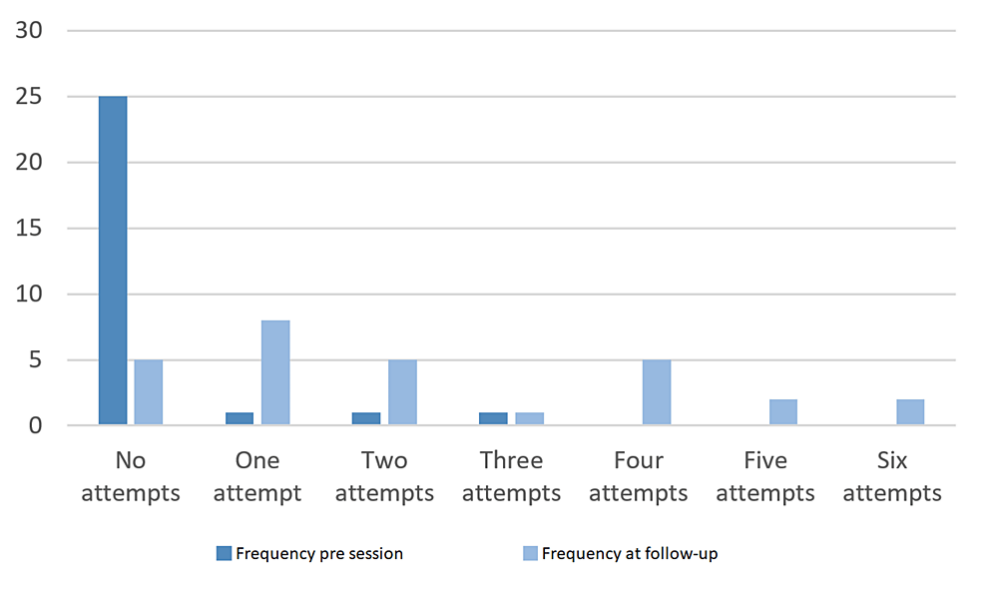
Number of FY1s who reported a given number of attempts at US-guided venous cannulation in the month before the teaching session and the month prior to follow-up.

Additionally, the number of FY1s who attempted ultrasound-guided venous cannulation at least once was vastly increased, from 10% in September to 82% in December (Figure 3).

Escalations to seniors

**Figure 4:**
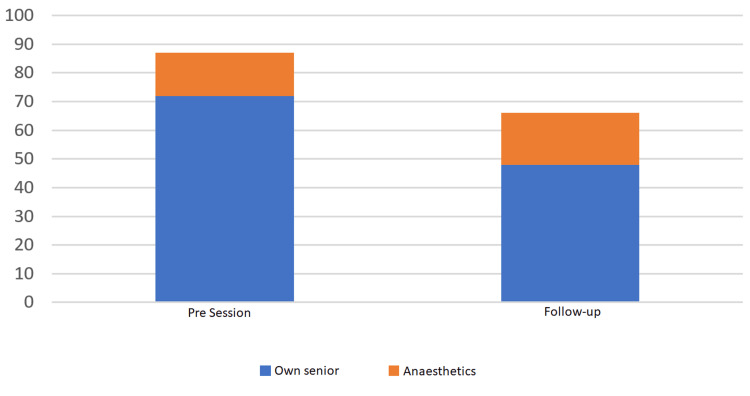
Number of times FY1s reported escalating a cannula to their own senior (blue) and anaesthetics (orange) in the month before the teaching session and the month prior to follow-up.

There was a notable decline in the number of cannulas FY1s escalated to their own senior between September and August, from 72 to 48 (33.33% decrease). However, there was little change in the reported number of calls to the on-call anesthetist, from 15 to 18 (20% increase) (Figure 4).

Success rate

**Figure 5:**
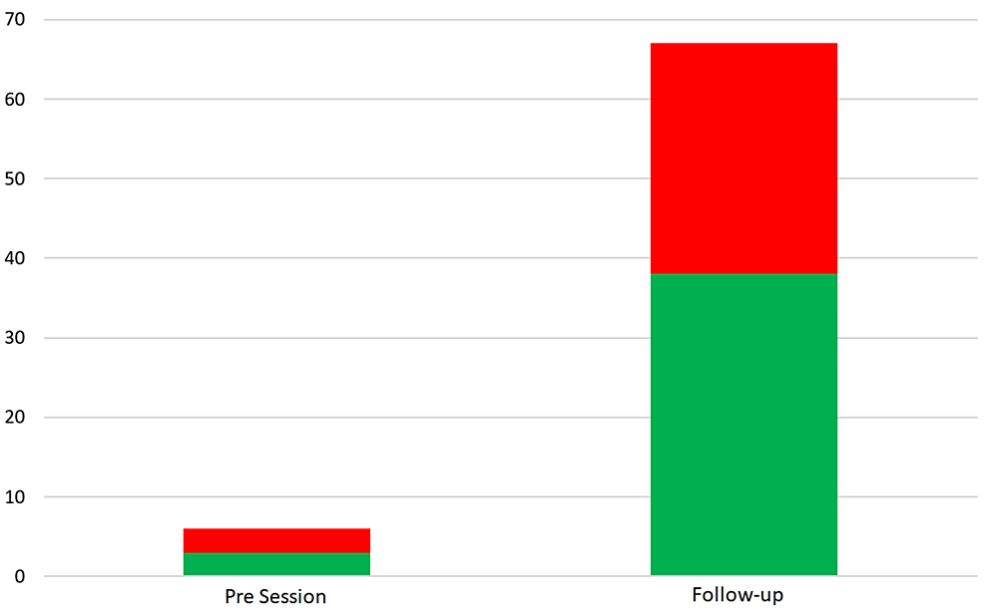
Bar chart showing successful (green) and unsuccessful (red) attempts at ultrasound-guided venous cannulation in the month before the teaching session and the month prior to follow-up.

There was a modest increase in the reported success rate with 50% (3/6) success in September compared to 60% (38/63) in December (Figure 5).

## Discussion

This study’s strongest finding is that near-peer teaching of ultrasound-guided venous cannulation produced FY1s who feel relatively confident in the skill, and who attempt it with some frequency (over two attempts per month per FY1). The study finds a 10-fold increase in the number of attempts at ultrasound-guided venous cannulation being made, a sustained improvement in confidence in the skill over a three-month period, and a clear increase in success rate. These findings are in keeping with previous literature relating to the usage of ultrasound-guided venous cannulation by FY1s [13,14].

These all indicate that the approach of providing an hour-long teaching session delivered by near-peers can be a highly effective way to introduce FY1s to ultrasound-guided venous cannulation. True expertise in the skill can clearly only be achieved through years of practice and a detailed understanding of ultrasound physics. However, it seems that the hour-long session has allowed FY1s to take a first step along the path to expertise by enabling them to gain practice and feedback on the wards and providing them with a skill that is of great use in their current clinical practice.

Alongside these findings, a large decrease in the number of times FY1s have had to escalate cannulas to senior colleagues has been identified. The impact of this likely includes patients receiving medications more quickly and reducing the impact on services from senior clinicians being pulled away to gain IV access. It is worth noting that FY1s would be expected to develop their abilities in cannulation and therefore have to call for help less often over the course of three months, even without ultrasound. Nevertheless, it would be a reasonable assumption that many of the successful ultrasound-guided venous cannulations would have otherwise been escalated to seniors if the FY1s had not had the training.

There was, however, no identified reduction in calls to anesthetics. Other studies have stated that FY1s believed their skills with ultrasound would result in fewer calls to anesthetists [14], but this appears to be the first study to attempt to quantify this. The authors were surprised not to find a reduction here.

A plausible explanation for this could be that the FY1s have achieved a basic level of competence with ultrasound which allows them to gain vascular access in patients with a low or medium level of complexity. However, they may not be able to successfully cannulate patients with very difficult veins even with ultrasound, necessitating a call to the on-call anesthetist. A more thorough audit of this issue, for example, via an anesthetic bleep audit before and following a similar teaching session next year, could provide valuable data to better understand this issue.

Limitations

The most obvious limitation of this study is relying on retrospective self-reporting of venous cannulation attempts and escalations. This means it is highly unlikely the results are precise. However, it was felt that the data would be accurate enough to permit analysis - the trends would hold true even if the precise figures were slightly inaccurate. Furthermore, alternative methods of data collection (such as daily surveys or observational methods) would seem disproportionate and unlikely to be feasible.

This study also lacks a control group. This would have given useful data as a reduction in calls to seniors would be expected over the course of three months as FY1s improve their cannulation skills, but it is difficult to quantify how large a reduction to expect. Some increase in the usage of ultrasound-guided venous cannulation could also be expected even without the teaching intervention, due to helpful senior colleagues demonstrating the skill on the ward - although the ten-fold increase this study identified would feel unlikely. Again, this was considered but it was felt it would be unreasonable to refuse to teach the skill to a number of the FY1s for this purpose.

## Conclusions

The aim of this teaching intervention was to help newly qualified FY1s doctors develop their skills in ultrasound-guided venous cannulation. This aim was met, with the FY1s using the skill much more frequently and more successfully. Further research would be required to confirm the impact on calls to senior clinicians, though it seems likely that these would be reduced. These aims were achieved via an hour-long introduction to ultrasound-guided venous cannulation, delivered by near peers. This therefore appears to be an effective model through which to teach the skill. Ultrasound-guided venous cannulation will continue to be taught at our Trust in future years.
